# Effect of training and structured medication review on medication appropriateness in nursing home residents and on cooperation between health care professionals: the InTherAKT study protocol

**DOI:** 10.1186/s12877-017-0418-3

**Published:** 2017-01-18

**Authors:** Angelika Mahlknecht, Nadja Nestler, Ulrike Bauer, Nadine Schüßler, Jochen Schuler, Sebastian Scharer, Ralf Becker, Isabel Waltering, Georg Hempel, Oliver Schwalbe, Maria Flamm, Jürgen Osterbrink

**Affiliations:** 1 Institute of Nursing Science and Practice, Paracelsus Medical University, Strubergasse 21, 5020 Salzburg, Austria; 2 Institute of General Practice, Family Medicine and Preventive Medicine, Paracelsus Medical University, Strubergasse 21, 5020 Salzburg, Austria; 3 Hausärzteverbund Münster/Association of general practitioners of Muenster, Metzer Strasse 59, 48151 Muenster, Germany; 4 Institut für Pharmazeutische und Medizinische Chemie der Westfälischen Wilhelms-Universität Münster/Institute of Pharmaceutic and Medical Chemistry of the Westfaelische Wilhelms-University, Corrensstraße 48, 48149 Muenster, Germany; 5 Apothekerkammer Westfalen-Lippe/Chamber of Pharmacists Westphalia-Lippe, Bismarckallee 25, 48151 Muenster, Germany

**Keywords:** Medication review, Medication safety, Safety of drug therapy, Nursing home care, Long-term treatment, Medication appropriateness in nursing homes, Interprofessional

## Abstract

**Background:**

Pharmacotherapy in residents of nursing homes is critical due to the special vulnerability of this population. Medical care and interprofessional communication in nursing homes are often uncoordinated. As a consequence, polypharmacy and inappropriate medication use are common and may lead to hospitalizations and health hazards. The aim of this study is to optimize communication between the involved professional groups by specific training and by establishing a structured medication review process, and to improve medication appropriateness and patient-relevant health outcomes for residents of nursing homes.

**Methods/Design:**

The trial is designed as single-arm study. It involves 300 nursing home residents aged ≥ 65 years and the members of the different professional groups practising in nursing home care (15–20 general practitioners, nurses, pharmacists). The intervention consists of interprofessional education on safe medication use in geriatric patients, and a systematic interprofessional therapy check (recording, reviewing and adapting the medication of the participating residents by means of a specific online platform). The intervention period is divided into two phases; total project period is 3 years.

Primary outcome measure is the change in medication appropriateness according to the Medication Appropriateness Index. Secondary outcomes are cognitive performance, occurrence of delirium, agitation, tendency of falls, total number of drugs, number of potentially dangerous drug-drug interactions and appropriateness of recorded analgesic therapy regimens according to the Medication Appropriateness Index.

Data are collected at t_0_ (before the start of the intervention), t_1_ (after the first intervention period) and t_2_ (after the second intervention period). Cooperation and communication between the professional groups are investigated twice by qualitative interviews.

**Discussion:**

The project aims to establish a structured system for monitoring of drug therapy in nursing home residents. The newly developed online platform is designed to systematize and to improve the communication between the professional groups and, thus, to enhance quality and safety of drug therapy. Limitations of the study are the lack of a control group and the non-randomly recruited study sample.

**Trial registration:**

DRKS Data Management, DRKS-ID: DRKS00007900

## Background

Prescription and monitoring of drug therapy in older patients is a special challenge for all involved professional groups and requires attentive consideration about the expected benefit and potential harm. Older persons are more frequently exposed to adverse drug events (ADEs) because of age-related changes in body composition and function, and due to a higher frequency of multiple comorbid conditions [1]. Evidence regarding medical treatment in multimorbidity is scarce. Clinical recommendations and guidelines usually address single diseases; however, a concomitant use of several drugs (polypharmacy) in multimorbid older patients is common [2, 3] and in line with an increased risk of medication errors, inappropriate medication [4, 5] and ADEs [6].

A particularly challenging field is the surveillance of pharmacotherapy in institutions of inpatient care of older persons. Residents of nursing homes are considered an especially vulnerable population due to frequent physical, cognitive and sensory impairments and complex disabilities [7, 8]. In Germany, as in other European countries, regular review and adjustment of medication is not ensured in many institutions [9, 10]. GPs usually visit their patients living in nursing homes regularly, but patients are also visited and prescribed drugs by different specialists e.g. in internal medicine, geriatrics or geriatric psychiatry. Pharmacists generally dispense the medication, control the storage of drugs in nursing homes and train the nurses in adequate delivery of drugs. Additionally, pharmacists perform analyses of drug-drug interactions. They are, however, not consulted on a regular basis for complete medication reviews in German nursing homes. They are legally obliged to ensure that the provision and storage of drugs and medical products is adequate [11]. The role of the nurses in the medication process comprises delivery of drugs and monitoring of the residents’ clinical condition. In general, it is common to have several different caregivers in nursing homes, and regular interaction and coordination between health care professionals is not provided [12]. As a consequence, monitoring of pharmacotherapy is often insufficient and several studies have highlighted the demand of improving medication safety for nursing home residents [13–19]. A US study showed an ADE incidence of 9.8% per month with 28% of the detected ADEs being severe or lethal. Of the latter, 61% were classified as preventable [13]. According to estimation by the World Health Organization (WHO) [14], up to 10% of all hospitalizations can be attributed to avoidable ADEs; in older patients this number rises to 24% [6, 15]. Polypharmacy, medication errors and inappropriate medication use are common causes of ADEs and are particularly frequent in nursing home populations [16, 17], who are also potentially oversupplied with certain groups of substances such as neuroleptics or antidepressants [18, 19].

To address this issue, various studies have investigated the utility of structured medication review processes. Outcome measures were heterogeneous and results varied widely: on the one hand studies showed significant improvements in medication appropriateness measured by the Medication Appropriateness Index (MAI) [8] and Beers criteria [20] as well as significant improvements in number of drug-drug interactions [20] and number of drugs [12, 20]. Other studies found no effects on number of drugs, costs or on medication appropriateness according to Medical Product Agency guidelines (which represents a country-specific Swedish list of drugs with possible effects of cognitive impairment that was used before the introduction of Beers criteria or generally applicable PIP lists) [7, 10]. However, combined interprofessional approaches consisting of educational training and structured medication review processes [8, 21, 22] seem to be most promising.

Regarding patient-related outcomes, the only observed significant change consisted of a reduction in falls [10] while no changes in mortality [10, 12], residents’ behaviour [8], hospitalization rates, functional status or cognitive skills [10] have been noted so far. Nevertheless, it seems plausible that a structured medication plan with low probability of medication errors and a consequent surveillance does result in better patient outcomes. We therefore assume that a structured and consistent approach focusing on interprofessional communication and teamwork can benefit the health status of nursing home residents.

The aim of the study InTherAKT (“Initiative zur Therapiesicherheit in der Altenhilfe durch Kooperation und Teamwork”—Initiative for medication safety in nursing home care by means of cooperation and team work) is to improve safety of drug therapy for residents of nursing homes in Muenster (Germany). This shall be achieved by the implementation of the InTherAKT intervention, which combines specific training of the participating professional groups based on the blended learning concept as well as systematic recording, reviewing and adapting of medication of the participating residents by means of the InTherAKT- online Platform (I-oP). Thus, cooperation and communication processes between the professional groups involved in drug use shall be optimized and structured documentation and reflection of drug treatment shall be established.

### Study hypothesis

The InTherAKT intervention improves the appropriateness of medical prescriptions for nursing home residents (≥65 years old).

We hypothesize further that the InTherAKT intervention enhances the interprofessional cooperation between the participating professional groups and leads to positive outcomes for the included nursing home residents regarding their cognitive skills, by increasing their mobility and by reducing agitation as well as the probability of developing a delirium. Furthermore, we expect an effect of the intervention on the total number of prescribed drugs, a reduction of the number of potentially dangerous drug-drug interactions and increased appropriateness of the recorded analgesic drug regimens.

## Methods/Design

**Fig. 1:**
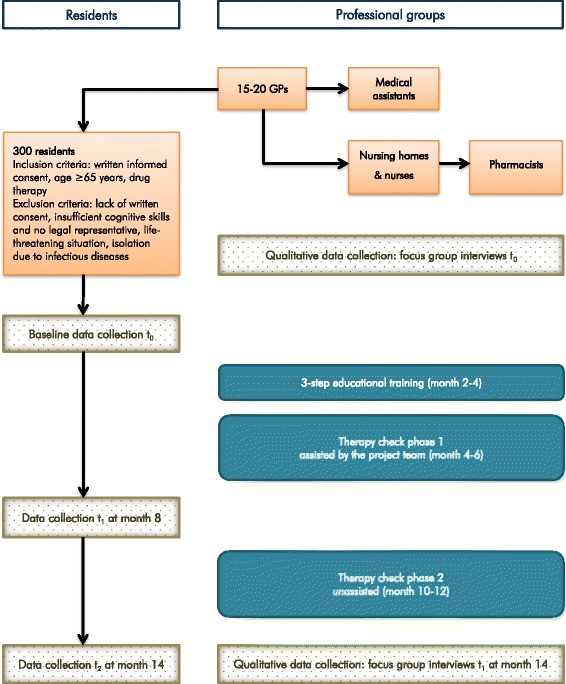
Study flow chart: recruitment phase and observational period

The InTherAKT project is a longitudinal, single-arm trial. The duration of the project is 3 years (June 2014 to July 2017), including planning, recruitment, data collection (see Fig. 1) and evaluation.

### Setting

The study is performed with members of the professional groups involved in inpatient care of older persons (general practitioners, nurses, pharmacists serving nursing homes, medical assistants and pharmacy technicians) and with residents of nursing homes in Muenster, North Rhine-Westphalia, Germany.

### Recruitment, study population, sample size

Based on experiences of recent studies in a similar setting [10, 22], the participating professional groups and residents of nursing homes are recruited successively. General practitioners (GPs) of the “Hausärzteverbund Münster” (association of GPs of Muenster), who hold a contract for intensified treatment of nursing home patients,^1^ are recruited primarily. In a second step all other GPs of the association are invited to participate. All patients living in nursing homes fulfilling the inclusion criteria are recruited via the respective GPs; residents and/or their legal representatives are informed and invited to participate by their GPs. Concurrently the nursing homes where the recruited residents live are invited to participate by the project team. In every nursing home, several nurses are nominated as responsible persons for the InTherAKT project. Finally, all pharmacists who have a contract with the respective nursing homes are invited to participate.

GPs are first sent written information by mail, then they are invited to a joint information meeting; further recruitment is carried out by personal visits of GPs who are not able to attend the information meeting. The residents, nursing homes and pharmacists are also visited personally by the study team to provide them with detailed written information about the project. GPs receive a financial incentive (20€/patient/quarter year) in order to increase participation.

Inclusion criteria for residents: written informed consent of the resident or of the legal representative, age ≥ 65 years, on pharmacotherapy (≥1 prescription/s).

Exclusion criteria for residents: missing declaration of consent, insufficient cognitive performance for making independent decisions and no legal representative, age < 65 years, acute life-threatening situation, isolation due to acute infections or multiresistant micro-organisms.

Inclusion criteria for professional groups: GPs with patients in nursing homes, pharmacists supplying nursing homes, trained medical assistants and nurses with 3 year or more state-approved training.

Power calculation was performed with a power of 0.8 (1-β = 80%). For the primary endpoint (see below), according to comparable trials [23] an effect size of at least 0.33 can be assumed. After considering possible reduction factors for the sample size (exclusion criteria, refusal of participation, death during the study period, cognitive function, unusable data sets) and ensuring the possibility of subgroup analyses, the size of the gross sample results in 300 residents.

### Intervention

The InTherAKT intervention aims to improve three processes: building competences, changing the mode of communication and promoting and structuring interprofessional cooperation. It is standardized as far as possible and consists of a profession-specific knowledge building about medication processes, ADEs and drug risk management and the introduction of a newly developed communication software, the InTherAKT- online Platform (I-oP).

*Knowledge building*: The training concept is developed and executed by a multiprofessional group, consisting of members of the project team: one general practitioner, one specialist in internal medicine with expertise in polypharmacy, two clinical pharmacists and one master of nursing science (in general, a German master of nursing science is an advanced-level postgraduate degree for registered nurses; the master of nursing science in our project team was practising as a nurse in the past and is currently working as research associate). The delivery mode of the training follows the blended learning concept [24], which combines face-to-face teaching with computer technology (online-training sessions in our case). Training tools are developed in a multiprofessional discussion and are based on current evidence [25–42] and guided by the individual challenges of each professional group and by the obstacles and risks arising in course of interprofessional cooperation. The following topics are covered (all related to the special characteristics and needs of older and multimorbid patients): particularity of drug therapy in older adults, pharmacokinetics and pharmacodynamics, drug-drug and drug-disease interactions, medication process and medication errors, ADEs and risk groups, monitoring of ADEs, risk analysis, pharmacovigilance, polypharmacy, potentially inappropriate prescriptions (PIP), PIP lists, over- and underprescribing, deprescribing and prioritizing of medications, medication review, legal aspects of drug therapy and strategies to enhance multiprofessional cooperation. Training combines three main steps:

- ▪ A starting on-site workshop for all participating professional groups together (duration: 3 h).
- ▪ Afterwards, three profession-specific online training sessions are performed by each professional group (duration: 30–45 min each) with problem-oriented case handling. During a period of 5 weeks, participants are invited to access the audio-visual presentations on the online area of the Paracelsus Medical University and to work through three patient case files addressing medication-related problems in older adults.
- ▪ In a final on-site event (duration: 1.5 h), the case solutions are addressed by all professional groups together and possible conclusions on aspects of cooperation improvement are drawn. The strategies of improved cooperation are based on the results of the initially conducted group interviews (see below) as well as on best practice models [22].

**Fig. 2:**
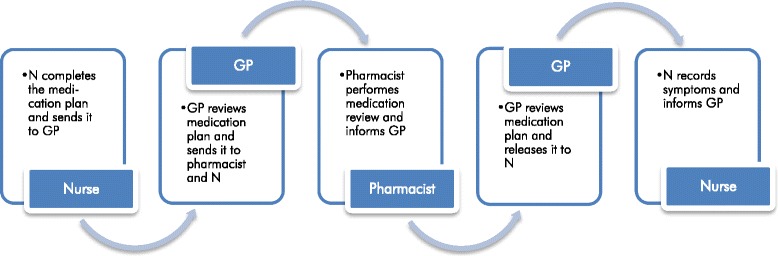
Therapy check process via the InTherAKT- online Platform (I-oP)

*Therapy check*: the InTherAKT—online Platform (I-oP) is intended to be the key instrument for optimizing the communication and cooperation between the professional groups. The I-oP is involved in the whole medication process in the participating facilities. It enables the execution of a dedicated therapy check process, consisting of six steps (see Fig. 2): (1) Collection and entering of resident’s data and current medication by the nurse in an electronic data sheet, which is based on the German national medication plan version 2.0^2^ [43]. (2) Completion, review and authorization of the medication plan by the GP and release to the nurse. (3) In a next step, the medication is reviewed by the pharmacist: medication review^3^ type 1 (simple medication review) [44, 45]. (4) The GP reviews the suggestions of the pharmacist and releases the revised medication plan to the nurse. The GP informs the resident about any changes in the medication. (5) Surveillance: during the therapy check process, the nurse records any new symptoms of the residents in an online therapy monitoring form within the I-oP. The therapy monitoring form has been developed by another German research team (Thürmann et Jaehde) [22]. The purpose of this monitoring is to detect any new symptoms possibly related to changes of the medication and to respond rapidly (the nurse informs the responsible GP about any abnormal findings via the I-oP). Causality regarding ADEs is not explicitly assessed. The following categories of symptoms are recorded within the monitoring form: potentially allergic reactions, bleeding, gastrointestinal problems, neurological problems (e.g. vertigo, coordination disturb), cardiovascular problems (e.g. edema, hypotension), psychiatric problems (e.g. confusion, delirium); additionally, any changes in medication, hospital visits or physician’s consultations are recorded. Therapy monitoring is initially planned on a daily basis for 3 weeks, thereafter once a week. (6) Case conferences are intended to take place on demand, when interprofessional decision-making is required; they may be convened at any step of the process. Contents and agreements of the case conferences are recorded in the I-oP.

Communication between GPs, nurses and pharmacists is conducted via a standardized messaging system within the I-oP. Messages are generated automatically by the platform whenever a step of the therapy check is completed and are sent to the email address of the concerning GP, nurse and/or pharmacist. Every health care professional uses the online platform whenever he/she will be advised that there is a task to perform. There are no determined time-slots. After completing the therapy check process, the nurses continue to use the I-oP at least once a week for therapy monitoring. GPs and pharmacists continue to check the software every time they are advised. In cases of urgent need for action (e.g. acute or severe symptoms), communication between health care professionals is conducted via telephone and the I-oP is used for documentation. The complete therapy check procedure (Fig. 2) is performed at least once during each intervention period and as soon as a medication is changed or added.

The intervention period is divided into two phases with a different extent of assistance of the project team (see Fig. 1):

Phase 1 in the period between t_0_ and t_1_ (month 4–6): the therapy check is accompanied by the project team who supervises the activities from the background and provides assistance if needed.

Phase 2 between t_1_ and t_2_, unassisted intervention period (month 10–12): process of autonomous cooperation of the participating professional groups, based on the experience of the first intervention phase. The project team provides assistance if no activities are recorded within the I-oP.

### Outcome measurements

Two different methodological approaches will be applied for measuring outcomes: resident-specific outcomes (see below: primary endpoint, secondary endpoints) are investigated quantitatively by means of standardized record sheets and tests performed by trained study assistants; professional group related outcomes (changes in cooperation and communication between the caregivers involved in the medication process of nursing home residents) are assessed by guided group interviews (qualitative research). As a base for the interviews, a manual (guideline) was developed by the project team comprising questions like: “How do you experience cooperation and communication with the other professional groups regarding safety of drug therapy?” “Which function do you think you hold to ensure safety of drug therapy?” “What should be changed in the cooperation in order to achieve optimized results?” The interviews are conducted before and after the intervention period by the project team in three sessions: 1. interview with GPs and medical assistants, 2. interview with nurses, 3. interview with pharmacists and pharmacy technicians. All interviews are digitally recorded, transcribed and analyzed (see below) by the project team.

*Primary endpoint*: Change in the appropriateness of *all* prescribed medications, measured according to the MAI [23].

#### *Secondary endpoints*

- ▪ Cognitive performance, measured according to the Mini Mental State Examination (MMSE) [46–48] and Dementia Screening Scale (DSS) [49]
- ▪ Probability of developing delirium, measured according to the Delirium Observation Screening Scale (DOS) [50]
- ▪ Agitation in residents with MMSE < 18 [51], measured according to the Cohen Mansfield Agitation Inventory (CMAI-D) [52]
- ▪ Mobility and tendency of falls, measured according to the Timed Get Up and Go (TUG) test [53]
- ▪ Total number of drugs
- ▪ Number of potentially dangerous drug-drug-interactions, measured according to the Up-to-Date/Lexicomp database [54]
- ▪ Appropriateness of recorded analgesics, measured according to the Medication Appropriateness Index (MAI) [23].

### Data collection procedures

Resident-related data are collected by study assistants via a collection tool for tablet PCs within an electronic case report form at three points in time (see Fig. 1): t_0_ after obtaining written informed consent and before starting the intervention, t_1_ after the first intervention period (month 8), t_2_ after the second intervention period (month 14). Training of study assistants and data assessment is carried out by the project team.

#### Quantitative standardized data collection for the endpoints

Parameters collected for the MAI at t_0_, t_1_ and t_2_ according to the documentation of the nursing homes (calculation of the MAI score by clinical pharmacists; further analysis of the MAI Score by the project team):

- Height, weight, age, current diagnoses (ICD-10 coded)
- Complete list of medication including brand names and International Nonproprietary Names (INN) of drugs, indication, dosage, mode of application, duration of therapy, instructions for application, known drug intolerances
- Creatinine, potassium, blood sugar, HbA1c, LDL-cholesterol according to documentation of the nursing homes or to documentation of the GP surgery (if applicable)
- Presence of pain and pain intensity: by means of the Verbal Rating Scale (VRS) [55] in case of a MMSE-score of 30–18 points. If the MMSE-score amounts below 18 points, the external assessment instrument BESD (“Beobachtung von Schmerz bei Demenz”, the German version of Pain Assessment in Advanced Dementia Scale, PAINAD) [56, 57] with standardised mobilizations will be applied in addition to the MMSE.

For the secondary outcomes, the following assessments and tests are performed with the residents at t_0_, t_1_ and t_2_: MMSE, DSS, CMAI-D, DOS, TUG test (see above).

Study assistants pseudonymize all participant residents by means of ID allocation. Data are accessible to the project team and exportable for analysis only after pseudonymization.

#### Control variables

To describe the intervention phases, the following resident data are collected according to the documentation of the nursing homes:

- Level of care and degree of physical function (Barthel scale) [58], used with the Hamburg Classification Manual [59]: at t_0_, t_1_ and t_2_.
- Number of GP consultations, consultations of an emergency physician, hospitalizations, falls, performed case conferences during the intervention period and mortality: at t_1_ and t_2_.

#### Therapy check via the I-oP

The following data are registered in the I-oP for the therapy check procedure: name of nursing home resident, date of birth, sex, diagnoses, medication list (brand name and INN, indication, dosage, form of administration, time of administration, indication, regular/as-needed medication, known drug intolerances and administration problems) and clinical symptoms of the resident. The platform is electronically secured, data are only available by individual access keys. The recorded data are stored in the I-oP for the complete duration of the project.

#### Professional groups and structural data

Qualitative data acquisition is performed before t_0_ and after t_2_ (Fig. 1) by means of guideline-based group interviews with GPs, nurses, pharmacists, medical assistants and pharmaceutical technicians to assess any changes in quality of cooperation and communication between the individual professional groups.

In the course of the interviews, the following quantitative data of the participating professional groups are collected: sex, time spent in the profession/care facility/surgery, qualification standard, advanced training in medication safety and extent of training.

Structural data of the participating institutions are collected by means of a structure assessment sheet and include:

- Nursing homes: number of residents, number of nurses and nursing assistants, number of supervising GPs, medical specialists and pharmacists, public or private institution.
- GP offices: single-handed or group office, average number of patients per quarter, number of supervised residents of nursing homes.
- Pharmacies: type of pharmacy, number of employees and pharmacists, specialisations or certifications (medication safety, geriatric pharmacy, community pharmacy).

The structure assessment sheet is filled in at t_0_, while at t_1_ and t_2_ the institutions will be questioned about any changes.

### Data evaluation and statistical analysis

Outcome data are collected and stored via a collection tool for tablet PCs within an electronic case report form. After collection, data are pseudonymized and imported into the IBM^©^SPSS statistical programme for analysis. The number of convened case conferences is imported from the I-oP.

For the quantitative part of the study, procedures of descriptive and analytical statistics are used. The focus will lie on comprehensive descriptions of the collected data for the different times of collection. Baseline and demographic characteristics are analyzed in a descriptive way (number of valid cases, parameters of central tendency, dispersion and distribution depending on quantitative or qualitative variables and number and proportions for qualitative variables); bi- and multivariate analyses are performed for subgroup analyses (based on association and correlation coefficients, e.g. Chi^2^, Spearman’s rho, Pearson’s r, simple and multiple linear regression). In addition to the descriptive comparison of results from the collection times inference-statistical procedures will be applied. The significance level is set to 0.05 (α = 5%) for all one- or two-sided significance tests. The primary endpoint, change in MAI after the intervention period, will be tested by using a t-test for dependent samples. For secondary outcomes, parametric procedures (t-test for dependent samples) and non-parametric procedures (Wilcoxon test) will be used where appropriate (dependent on quantitative or qualitative variables). Drop-out and loss of follow-up will be described. Due to the study’s strongly descriptive character the application of hypothesis-testing procedures primarily aims to generate hypotheses.

For the qualitative part of the study, the guideline-based group interviews are digitally recorded and then transcribed. The transcriptions are analysed by means of a coding system, which was developed by the project team according to the interview manual. All answers of the professional groups are itemized and categorized by use of these codes (e.g. paragraphs which describe quality of cooperation with GPs are divided into the following categories: good cooperation/problems in cooperation/occasions for cooperation). Coded paragraphs are filtered by use of a specific software for qualitative analysis (MAXQDA) and then paraphrased in order to obtain a fluent and homogeneous text. The last step consists in concentrating and summarizing the text and in drawing the main conclusions [60].

## Discussion

Medication safety and prevention of ADEs has become a field of special interest in medical and pharmaceutical care as well as in health policy. The recent “Aktionsplan des Bundesministeriums für Gesundheit zur Verbesserung der Arzneimitteltherapiesicherheit in Deutschland“ (action plan of the Federal Ministry of Health for improving medication safety in Germany) [61] recommends, among others, the use of electronic devices and strengthening of interprofessional communication as priority fields for applying strategies to improve safety of drug therapy. The project InTherAKT addresses these topics by implementing and testing an electronic tool for structured documentation and reflection of medication, which shall be established on a permanent basis and shall lead to improved communication between professional groups. We expect this will create a substantial precondition for improving medication safety in nursing homes. Additionally, the three-step training with two joint events for all participating professional groups shall reinforce the interprofessional communication and cooperation.

The therapy check process is conducted and documented electronically. Nurses perform documentation on the I-oP additionally to their primary documentation; they provide the required resident-related information and their insight to the GPs and pharmacists to initiate a dedicated review process. Furthermore, the nurses conduct monitoring of clinical symptoms and documentation of suspect drug-related problems. The pharmacists provide their expertise in the medication review process and hold a constructive, problem solving orientated dialogue with the GPs. The latter consider all suggestions and decide the final therapeutic strategies. The competence of the GP as responsible person for the therapeutic decision will be strengthened. The I-oP enables a short track communication between the different caregivers; additional time consuming personal contacts are required only for the case conferences. Thus, communication between nurses, GPs and pharmacists shall not only be improved but also facilitated.

The two intervention periods differ in the extent of support by the project team, in order to enable the study participants to continue the therapy check autonomously after the end of the project.

A special feature of the trial is the inclusion of both residents and professional groups in research by use of quantitative and qualitative methods. The primary outcome (medication appropriateness according to the MAI) is investigated by clinical pharmacists, who are not involved in the therapy check process.

Considering the experience of previous research in a similar setting [10, 22], we chose to start recruitment with GPs, as they are the central stakeholders in the medication process and participation of residents, nursing homes and pharmacists depends on GPs’ will to collaborate. One methodical weakness is that study participants do not represent a random sample, but rather a selection by choice. GPs of the local GP association and with a special contract for increasing visits and nursing home contacts are addressed primarily, because of a higher probability to achieve the aspired number of participating residents. This leads to a further limitation, the lack of a control group. The single-arm design has been chosen as the study is conducted within one city, which would entail the risk of contamination between intervention and control group as each GP has patients in several different nursing homes. Another limitation is that awareness of GPs about surveillance of their prescribing could influence prescribing behaviour beyond the expected impact of the intervention (study effect).

Moreover, regarding the primary endpoint, it has to be noted that we do assess all drugs including PRN medications as *prescribed*; it would be interesting to evaluate all drugs as administered, however, this is not scope of the present study.

## Trial status

At the time of submission of the manuscript in January 2016, recruitment, focus group interviews, baseline data collection and training of the participating professional groups have been concluded and the first therapy check phase is ongoing.

## Abbreviations

ADE(s): Adverse drug event(s)
BESD: Beobachtung von Schmerz bei Demenz, German version of PAINAD (see below)
CMAI-D: Cohen Mansfield Agitation Inventory (German version used)
DOS: Delirium Observation Screening Scale
DRKS: German Clinical Trials Register
DSS: Dementia Screening Scale
GP: General practitioner
ICD-10: International Statistical Classification of Diseases and Related Health Problems
INN: International Nonproprietary Names
I-oP: InTherAKT- online Platform
MAI: Medication Appropriateness Index
MMSE: Mini Mental State Examination
PAINAD: Pain Assessment in Advanced Dementia Scale
PCNE: Pharmaceutical Care Network Europe
PIP: Potentially inappropriate prescription
PRN medication: Pro re nata medication (administered in the prescribed dosage if needed)
TUG: Timed Get Up and Go test
VRS: Verbal Rating Scale
WHO: World Health Organization

## Footnotes

1 Contract for intensified treatment of nursing home residents: special contract of care, where GPs commit theirselves to visit their patients who live in nursing homes periodically and with determined frequency; additionally, GPs holding these contracts collaborate with a special care assistant nurse, who meets patients regularly and refers any news and problems to the respective GP.

2 The National medication plan version 2.0 is a standardized, structured document, which was developed in Germany in 2014 with the aim to improve patient safety. The medication plan 2.0 will be introduced in 2016 at a national level in order to provide to the patients complete and comprehensible information regarding their current medication, and in order to enhance the flow of information between all professional groups involved in the medication process.

3 Medication review: the Pharmaceutical Care Network Europe (PCNE) distinguishes three types of medication review: a simple medication review is based on medication history, an intermediate medication review comprises also patient’s information, and an advanced medication review considers additionally clinical information [44].
